# Microarray Analysis Reveals the Changes in Circular RNA Expression and Molecular Mechanisms in Mice With Ventilator-Induced Lung Injury

**DOI:** 10.3389/fphys.2022.838196

**Published:** 2022-03-10

**Authors:** Shengsong Chen, Jingen Xia, Qingyuan Zhan, Yi Zhang

**Affiliations:** ^1^Department of Pulmonary and Critical Care Medicine, China-Japan Friendship Hospital, Center of Respiratory Medicine, Beijing, China; ^2^Graduate School of Peking Union Medical College, Chinese Academy of Medical Sciences, Beijing, China; ^3^National Center for Respiratory Medicine, Beijing, China; ^4^Institute of Respiratory Medicine, Chinese Academy of Medical Sciences, Beijing, China; ^5^National Clinical Research Center for Respiratory Diseases, Beijing, China; ^6^WHO Collaborating Centre for Tobacco Cessation and Respiratory Diseases Prevention, Beijing, China

**Keywords:** ventilator-induced lung injury, circRNAs, RNA sequencing, bioinformatics, circRNA-miRNA-mRNA interaction network

## Abstract

Circular RNA (circRNA) expression profiles in lung tissues from mice with and without ventilator-induced lung injury (VILI) were analyzed using high-throughput sequencing and bioinformatics to clarify their potential role in VILI pathogenesis and provide valuable molecular markers for VILI diagnosis and treatment. A VILI mouse model was established using high-tidal volume ventilation, and lung tissue was stained with HE and TUNEL. The present study used high-throughput sequencing technology to analyze the expression profile of circRNAs in the lung tissue of mice with and without VILI. Bioinformatics was used to analyze the enrichment of differentially expressed circRNAs using Gene Ontology and KEGG to predict function. Among the top 10 circRNAs with significant differential expression, we used real-time quantitative polymerase chain reaction technology (qRT-PCR) to verify the accuracy of the high-throughput sequencing results and constructed the corresponding circRNA-miRNA-mRNA-specific binding network map using software prediction. The most upregulated circRNAs were novel_circ_0000899 and novel_circ_0014815, and the most downregulated circRNAs were novel_circ_0015069. A total of 14,347 circRNAs were detected using high-throughput sequencing. Compared to the control group, 285 circRNAs were abnormally and significantly expressed in the lung tissues of VILI mice (|log2(FC)| > 1, *p* < 0.05). A total of 171 circRNAs were significantly upregulated, and 114 circRNAs were significantly downregulated. Gene ontology analyses indicated that the differentially expressed circRNAs were involved in multiple biological functions, such as regulation of metabolic processes, protein phosphorylation, and chromatin organization. KEGG pathway analyses revealed that the Ras signaling pathway, rap1 signaling pathway, PI3K − Akt signaling pathway, and ECM receiver interaction were related to the differentially expressed circRNAs. The qRT-PCR verification results were generally consistent with the circRNA expression trends of the high-throughput sequencing data. The circRNA-miRNA-mRNA interaction network suggested that miRNAs and mRNAs related to circRNAs played a key role in VILI. Differentially expressed circRNAs were identified in the tissues of VILI mice using high-throughput sequencing combined with bioinformatics analysis, and the results lay a foundation for further study of the mechanism of circRNAs in the occurrence and development of VILI.

## Background

Ventilator-induced lung injury (VILI) is caused or aggravated during the process of ventilator treatment (Chen et al., 2018). The main mechanism is ventilator-induced repeated alveolar hyperextension and collapse, also known as barotrauma, volume injury, and atelectasis (Chen et al., 2018). Ventilators directly cause lung injury but also cause distant organ dysfunction syndrome or a systemic inflammatory response (Curley et al., 2016). The mechanism may include mechanical stretching that triggers a cascade amplification reaction of activated lung proinflammatory factors and inflammatory cells, reactive oxygen species generation, and complement activation (Curley et al., 2016). The overexpression of inflammatory mediator damages lung tissue and causes secondary damage, which is defined as biotrauma (Curley et al., 2016). However, the potential molecular mechanisms of ventilator-mediated inflammation have not yet been clarified completely and effective treatment is lacking.

Circular RNAs (circRNAs) are a newly identified special type of noncoding RNA (ncRNA), whose length are greater than 200 nt, and they are primarily produced from pre-mRNA *via* variable splicing. (Li et al., 2018; Kristensen et al., 2019; Chen, 2020). The 5′ and 3′ ends of circRNA covalently bind to form a closed structure, which widely exists in animals. The special ring structure of circRNA enables it to escape RNase degradation and enhances its stability *in vivo* (Li et al., 2018; Kristensen et al., 2019; Chen, 2020). In recent years, the effects and action mechanisms of circRNAs in different diseases, including diabetes mellitus, neurological disorders, cardiovascular diseases, and cancer, have been reported (Li et al., 2018; Kristensen et al., 2019; Chen, 2020). In addition, circRNA biogenesis and function have also been explored in lung injury. For example, circBbs9 promoted PM2.5-induced lung inflammation in mice *via* NLRP3 inflammasome activation (Li et al., 2020). CIRC3P1 inhibited the production of proinflammatory cytokines and apoptosis in septic lung injury by regulating miR-21 (Jiang et al., 2020). Mechanical ventilation (MV) is an essential life support for sepsis patients with acute respiratory distress syndrome. However, the potential role and mechanism of circRNAs in the occurrence and development of VILI remain unclear.

In this study, we established a VILI mouse model, detected the expression of circRNAs in the lung tissue of VILI mice, analyzed the specific expression of circRNAs and studied the characteristics of the circRNA expression profile. This study may provide a foundation to elucidate the mechanism of VILI, intervention targets, and early biological diagnostic markers, which have important theoretical significance and application value.

## Materials and Methods

### Animals

Specific pathogen-free male C57BL/6 mice (aged 8–10 weeks, weight 20 ± 2 g) were purchased from Beijing Sipeifu Biotechnology Co., Ltd., China. All mice were conventionally housed at 22 ± 2°C under a 12 h light–dark cycle with access to water and food *ad libitum*. All animal experiments were performed in compliance with the policies in the National Institutes of Health (NIH) Guide for the Care and Use of Laboratory Animals and were approved by the Experimental Animal Ethics Committee of China-Japan Friendship Hospital.

### Mouse Model of VILI and Sample Collection

**Figure 1:**
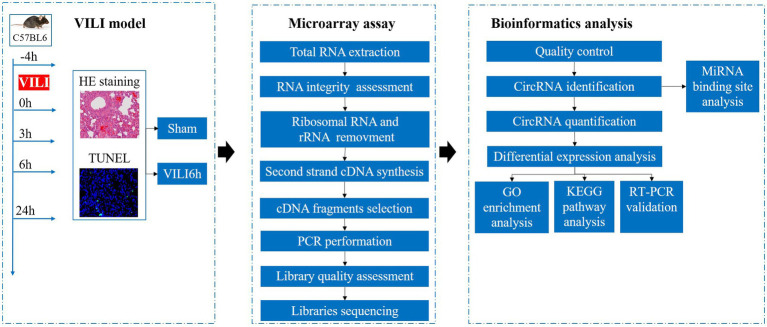
Analytical procedures used in this study.

Twenty-five mice were randomly divided into the sham group and the VILI groups. The experimental group received 1% sodium pentobarbital (100 mg/kg) as intraperitoneal anesthesia. Tracheotomy was performed, and a 22G trocar (BD Biosciences, United States) was inserted. The trachea was ligated to prevent air leakage, and continuous mechanical ventilation was performed for 4 h using a small animal ventilator (Harvard Apparatus, United States). During ventilation, the respiration and heart rate of the mice were closely observed, and the mice were kept warm and rehydrated. Sodium pentobarbital and rocuronium benzenesulfonic acid (0.6 mg/kg) were used as needed to maintain anesthesia and muscle relaxation, respectively. According to previous studies, we adopted a volume control mode for the ventilator, and the following appropriate parameters were set as: tidal volume 20 ml/kg, respiratory frequency 80/min, positive end-expiratory pressure 0 cm H_2_O, and fraction of inspiration O_2_ 21%. After 4 h, the ventilator was evacuated, the wound was sutured, and the mice were returned to the breeding cage after recovery from anesthesia. Samples were taken 0, 3, 6, and 24 h after ventilation. Mice in the sham group were intubated but not ventilated. The analytical procedure is shown in Figure 1.

### Histological Examination and TUNEL Detection

The lung tissue was fixed in 4% paraformaldehyde. The fixed tissue was dehydrated with alcohol, embedded, and sectioned. Nuclei were stained using hematoxylin, and the cytoplasm was stained with eosin. The pathological changes in lung tissue were observed under a microscope (Olympus, Japan). Two researchers without group information calculated the lung injury score, as described previously (Matute-Bello et al., 2011). Five independent variables, including neutrophils in the alveolar space, neutrophils in the interstitial space, the existence of hyaline membranes, proteinaceous debris filling the airspaces, and alveolar septal thickening, were used to generate a lung injury score.

Paraffin sections were deparaffinized in water and repaired with a working solution of proteinase K (Roche, Switzerland). The tissues were incubated in a working solution of lysis buffer and TdT and dUTP reagents (Roche, Switzerland). The nuclei were counterstained with DAPI (Roche, Switzerland), and the tissues were mounted on anti-fluorescence quenching mounting tablets. Images were captured under a microscope (Olympus, Japan). The sections were observed using a fluorescence microscope (Olympus, Japan). Ten fields of view were randomly selected, and the rate of cell apoptosis was determined using ImageJ (Bethesda, MD, United States). Green nuclei were considered positive apoptotic cells. Cells with blue nuclei were deemed normal, and the average value was determined accordingly. The ratio of the number of green cells to blue cells was the rate of cell apoptosis.

### Microarray Assay

Total RNA was extracted from lung tissue using TRIzol reagent (Invitrogen Life Technologies, Carlsbad, CA). RNA degradation and contamination were monitored on 1% agarose gels. RNA purity was confirmed using a NanoPhotometer^®^ spectrophotometer (IMPLEN, CA, United States). RNA integrity was assessed using the RNA Nano 6000 Assay Kit of the Bioanalyzer 2100 system (Agilent Technologies, CA, United States). A total amount of 5 μg RNA per sample was used as input material for the RNA sample preparations. First, ribosomal RNA was removed using an Epicenter Ribozero^™^ rRNA Removal Kit (Epicenter, United States), and rRNA-free residue was removed *via* ethanol precipitation. Subsequently, the linear RNA was digested with 3 U of RNase R (Epicenter, United States) per μg of RNA. The sequencing libraries were generated using the NEBNext^®^ Ultra^™^ Directional RNA Library Prep Kit for Illumina^®^ (NEB, United States) following the manufacturer’s recommendations. Briefly, fragmentation was performed using divalent cations under elevated temperature in NEBNext First Strand Synthesis Reaction Buffer (5×). First strand cDNA was synthesized using random hexamer primers and M-MuLV reverse transcriptase (RNaseH). Second strand cDNA synthesis was subsequently performed using DNA polymerase I and RNase H. In the reaction buffer, dNTPs with dTTP were replaced by dUTP. The remaining overhangs were converted into blunt ends *via* exonuclease/polymerase activities. After adenylation of the 3′ ends of DNA fragments, NEBNext adaptors with hairpin loop structures were ligated in preparation for hybridization. To preferentially select cDNA fragments 250–300 bp in length, the library fragments were purified using the AMPure XP system (Beckman Coulter, Beverly, United States). USER enzyme (3 μl, NEB, United States) was used with size-selected, adaptor-ligated cDNA at 37°C for 15 min followed by 5 min at 95°C before PCR. PCR was performed with Phusion High-Fidelity DNA polymerase, universal PCR primers, and Index (X) Primer. The products were purified (AMPure XP system), and library quality was assessed in an Agilent Bioanalyzer 2100 system. Clustering of the index-coded samples was performed using a cBot Cluster Generation System using TruSeq PE Cluster Kit v3-cBot-HS (Illumina) according to the manufacturer’s instructions. After cluster generation, the libraries were sequenced on an Illumina platform, and 150 bp paired reads were generated. Clustering of the index-coded samples was performed in a cBot Cluster Generation System using TruSeq PE Cluster Kit v3-cBot-HS (Illumina) according to the manufacturer’s instructions. After cluster generation, the libraries were sequenced in an Illumina platform, and 150 bp paired reads were generated. Quality control was performed after the raw data were obtained *via* sequencing. Mapping to the reference genome, circRNA identification, and quantification of gene expression levels were performed (see Supplementary Material). Differential expression analysis of two conditions/groups was performed using the DESeq R package (1.10.1). DESeq provides statistical routines for determining differential expression in digital gene expression data using a model based on the negative binomial distribution. The resulting *p* values were adjusted using Benjamini and Hochberg’s approach for controlling false discovery rates. Genes with an adjusted p value (padj or q value) found by DESeq were designated differentially expressed. Significantly differentially expressed circRNAs defined as having | log2(fold change) | > 1 and padj <0.05 were retained for further analyses.

### Gene Ontology and KEGG Pathway Analysis of Selected circRNAs

Gene Ontology (GO) enrichment analysis for host genes of differentially expressed circRNAs was implemented in the GOseq R package, in which gene length bias was corrected. GO terms with corrected values of *p* less than 0.05 were considered significantly enriched in differentially expressed genes. KEGG is a database resource for understanding the high-level functions and utilities of biological systems, such as cells, organisms, and ecosystems, from molecular-level information, especially large-scale molecular datasets generated by genome sequencing and other high-throughput experimental technologies.^1^ We used KOBAS software to test the statistical enrichment of differentially expressed genes or circRNA host genes in KEGG pathways. A pathway with a q value <0.05 was defined as being significantly enriched. We selected the 20 most significantly enriched pathways for display. If the enriched pathways were less than 20, all of them were displayed.

### Quantitative Reverse Transcription PCR Validation

We selected the most significantly expressed 10 circRNAs including seven upregulated and three downregulated circRNAs for verification, as shown in Table 1. Five samples from the experimental group and the control group were collected. Total RNA from lung tissue was extracted according to the TRIzol method (Invitrogen Life Technologies, Carlsbad, CA), and cDNA was synthesized using reverse transcription of RNA according to the instructions of the reverse transcription kit (TaKaRa, Japan). The SYBR fluorescent dye (TaKaRa, Japan) method was used for qRT-PCR detection, and β-actin was used as the internal control. The 2^−ΔΔCt^ method was used to calculate the relative expression of circRNAs in the sample. Table 1 lists the specific primers in this study.

**Table 1:** Top 10 differently expressed circRNA in microarray analysis.

**ID**	**VILI6h readcount**	**Control readcount**	**Log2FC**	**Pavl**	**Padj**
novel_circ_0000899	36.1076	7.2823	2.3121	3.42E-07	0.000485
novel_circ_0014815	135.7511	68.3372	1.0063	4.61E-07	0.000485
novel_circ_0004441	131.4399	66.571	0.9805	7.47E-07	0.000524
novel_circ_0016568	144.1706	75.1307	0.9417	1.85E-06	0.000802
novel_circ_0019935	47.3526	14.5784	1.7255	1.9E-06	0.000802
novel_circ_0007829	211.4379	131.2657	0.68975	3.08E-06	0.00108
novel_circ_0005213	70.1659	27.7482	1.3564	5.42E-06	0.001629
novel_circ_0015067	4.4282	26.5736	−2.6858	1.06E-05	0.002564
novel_circ_0015069	6.1445	29.5581	−2.3963	1.1E-05	0.002564
mmu_circ_0000098	13.7111	42.1314	−1.7319	1.68E-05	0.002564

### miRNA Target Prediction and Functional Enrichment Analysis

CircRNAs inhibit the function of miRNAs by binding to miRNAs. Therefore, miRNA binding site analysis of the identified circRNAs is helpful to further study the function of circRNAs. MicroRNA target sites in the exons of circRNA loci were identified using miRanda. MiRNA-mRNA networks were identified using miRanda software based on the total score value and energy value of all target prediction binding sites. Cytoscape software was used to construct the circRNA-miRNA-mRNA networks.

### Statistics

All experimental data are shown as the mean ± standard deviation. Student’s *t*-test was used to analyze the statistical significance of microarray data and qRT-PCR. One-way ANOVA was performed to compare the VILI 6 h group with the sham, VILI 0 h, VILI 3 h, and VILI 24 h groups. The statistical analyses were performed using Statistical Product and Service Solutions 19.0 (SPSS, Systat Software, San Jose, CA, United States). *p* < 0.05 was considered statistically significant.

## Results

### VILI Model Induced With High-Tidal Volume Mechanical Ventilation

**Figure 2:**
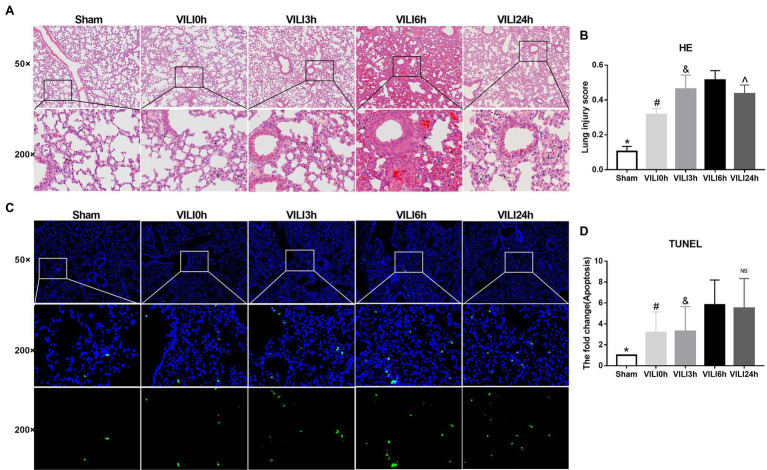
Identification of the VILI model induced by high-tidal volume mechanical ventilation. **(A)** HE staining of lung tissue in each group. Figure marker: →, macrophage; ^, alveolar hemorrhage; *, alveolar wall thickening; and &, Alveolar collapse. **(B)** Lung injury scores of lung tissue in each group, *n* = 3. **(C)** TUNEL staining of lung tissue in each group. **(D)** Apoptosis of lung tissue in each group after, *n* = 3. Bars represent means ± standard deviation. VILI. Ventilator-induced lung injury. HE. hematoxylin and eosin. TUNEL. TdT-mediated dUTP Nick-End Labeling. ^*^p < 0.05 VILI 6 h vs. sham, ^#^*p* < 0.05 VILI 6 h vs. VILI 0 h, ^&^*p* < 0.05 VILI 6 h vs. VILI 3 h, ^^^*p* < 0.05 VILI 6 h vs. VILI 24 h, and ^ns^ *p* > 0.05 VILI 6 h vs. VILI 24 h.

We constructed lung injury models at 0 h, 3 h, 6 h, and 24 h after the end of high-tidal volume mechanical ventilation. HE staining was used to evaluate lung injury (Figures 2A,B). The results showed that the structure of the lung tissue of the ventilation groups was significantly damaged compared to that of the sham group, the alveolar cavity was bleeding, and the thickness of the alveolar wall was increased significantly. Collapse and inflammatory cell infiltration were also observed. The most severe lung injury occurred 6 h after VILI (VILI 6 h) and was relieved 24 h after VILI (VILI24h). TUNEL staining was used to measure cell apoptosis. The results showed that the VILI 6 h and VILI 24 h groups had the highest ratio of cell apoptosis, and there was no difference between the two groups (Figures 2C,D). Our previous study also observed that the histological lesions were most severe in the VILI 6 h group (Zhang et al., 2019). Therefore, the VILI 6 h group was selected as the experimental group for further analyses.

### circRNA Expression Profiles

**Figure 3:**
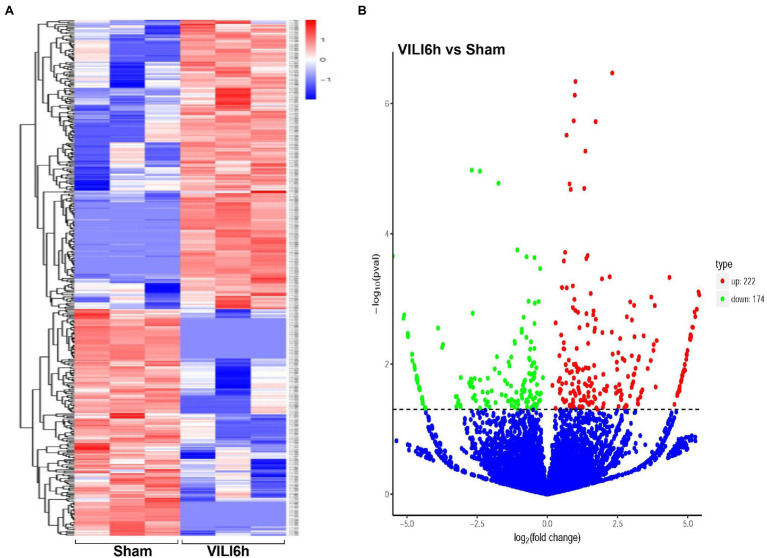
Differentially expressed circRNAs between VILI 6 h and sham mice. **(A)** Heat map of the differentially expressed circRNAs in VILI 6 h and sham mice. The X-axis represents samples, and the Y-axis represents differentially expressed genes. The genes are clustered according to the degree of expression similarity on the left, and each sample is clustered according to the similarity of expression profiles at the top. The expression is gradually upregulated from blue to red, and the number is the relative expression after homogenization. The red and blue colors indicate upregulation and downregulation, respectively. **(B)** Volcano plot for the differentially expressed circRNAs in VILI 6 h and sham mice. The X-axis represents the fold change of circRNA expression (log2 Fold Change) in different groups, and the Y-axis represents the statistically significant degree of circRNA expression change. The scattered dots in the figure represent each circRNA, the blue dots represent the circRNAs with no significant difference, the red dots represent the differential circRNAs that are significantly upregulated, and the green dots represent the differential circRNAs that are significantly downregulated. The dotted line represents *p* = 0.05. VILI. Ventilator-induced lung injury.

Microarray assays identified a total of 14,347 circRNAs from the VILI 6 h group and control group. A total of 945 circRNAs were identified previously, and 13,402 circRNAs were identified for the first time in this study. A total of 171 upregulated and 114 downregulated circRNAs were identified using the cutoff | log2(FC)| > 1 and padj <0.05. A volcano map (Figure 3A) was drawn using log2FC and -log10p. A heatmap was drawn according to the relative expression in each group (Figure 3B). Table 2 showed the most significantly expressed 10 circRNAs, including seven upregulated circRNAs and three down regulated circRNAs. The upregulated circRNAs were novel_circ_0000899, novel_circ_0014815, novel_circ_0004441, novel_circ_0016568, novel_circ_0019935, novel_circ_0007829, and novel_circ_0005213. The downregulated circRNAs were novel_circ_0015069, mmu_circ_0000098, and novel_circ_0015067.

**Table 2:** The primer list was used for real-time quantitative PCR.

**Gene name**	**Bidirectional primer sequences**	**The length of the product (bp)**
novel_circ_0000899	GATGCTGAGTGGCCCTGAG	155
	CAGAGACTGGTGTTGGGCTC	
novel_circ_0014815	TGCCTGGCTACGGGTTGTTT	253
	GCCTTCCGCATCTATGGTCT	
novel_circ_0004441	CACTGCCTGGGACAAAGATG	198
	TCGACCCTCAATCAAGGTGA	
novel_circ_0016568	AGAATGATGATGACCCACAG	158
	GTCCCAGAAGAAACTTGTAAAG	
novel_circ_0019935	AGACAGCTTCTTTCCCGTGG	149
	GCATGACCACCTAGCTCTCC	
novel_circ_0007829	TAACTGGTGGCAGACATCCC	172
	GTGGGTCTCCAGATAGAAGTGC	
novel_circ_0005213	CAGCAGCAAATGAGAGCCAC	168
	CACTCTCTGGTCACATCCCG	
novel_circ_0015067	CGGGCTCCTCATACTCCATC	91
	TGTCCTTCATCCCTCTTGCG	
novel_circ_0015069	AAAATCGCTGAGTACAAACGC	121
	TCCTGTTGATGGAGCTGACG	
mmu_circ_0000098	CTCTGAGTCACTAAGCGAGAA	212
	CCTGAGCCTACAGTAACAGC	
β-actin	GCACCACACCTTCTACAATG	262
	GTGAGGGAGAGCATAGCC	

### circRNA Gene Symbols and Pathway Analysis

**Figure 4:**
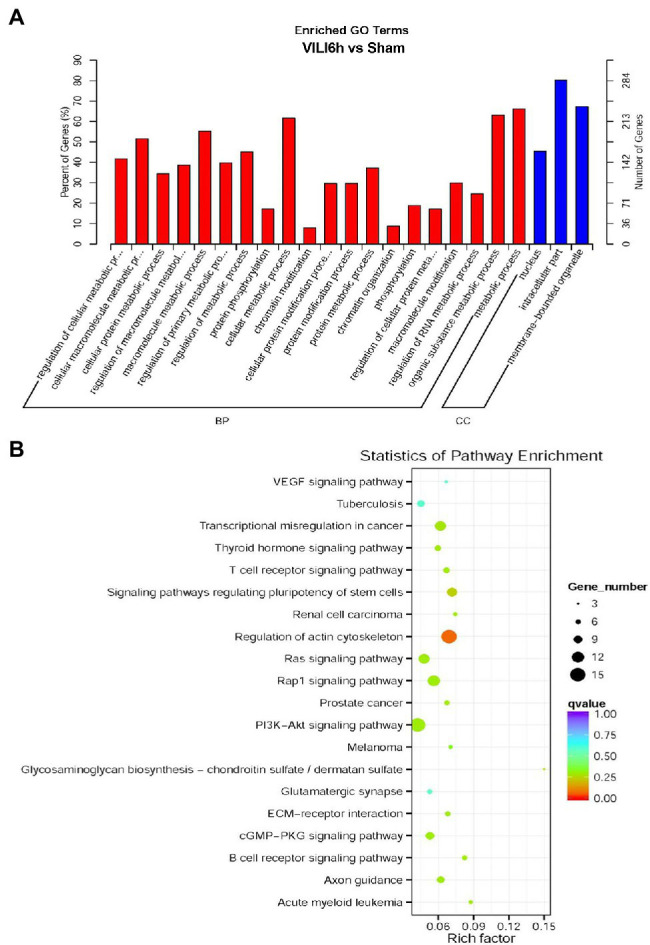
Gene Ontology (GO) and Kyoto Encyclopedia of Genes and Genomes pathway analyses results. **(A)** GO enrichment map of circRNA host genes. BP, biological process; CC, Cellular component. **(B)** The X-axis represents the rich factor, the Y-axis represents the name of the pathway, the size of the dot indicates the number of source genes in this pathway, and the color of the dot corresponds to different q value ranges.

Gene Ontology (GO) and KEGG enrichment analyses were used to analyze the differentially expressed circRNAs. GO annotation analysis (Figure 4A) of the differentially expressed circRNAs was used to classify the functions of these proteins at three levels: biological processes (BP), molecular function (MF), and cell component (CC). The differentially expressed circRNAs were primarily enriched in the following BP terms: regulation of cellular macromolecule and protein metabolic processes, protein phosphorylation, chromatin modification, protein modification processes, chromatin organization, phosphorylation, and regulation of RNA metabolic processes. The CC terms enriched in the differentially expressed circRNAs were nucleus, intracellular, and membrane-bounded organelle. KEGG analysis (Figure 4B) showed that the differentially expressed circRNAs were primarily enriched in the VEGF signaling pathway, thyroid hormone signaling pathway, T-cell receptor signaling pathway, regulation of actin cytoskeleton, Ras signaling pathway, rap1 signaling pathway, PI3K − Akt signaling pathway, glycosaminoglycan biosynthesis, the glutamatergic synapse, ECM − receptor interaction, cGMP−PKG signaling pathway, and B cell receptor signaling pathway.

### Validation of the Microarray Data Using qRT-PCR

**Figure 5:**
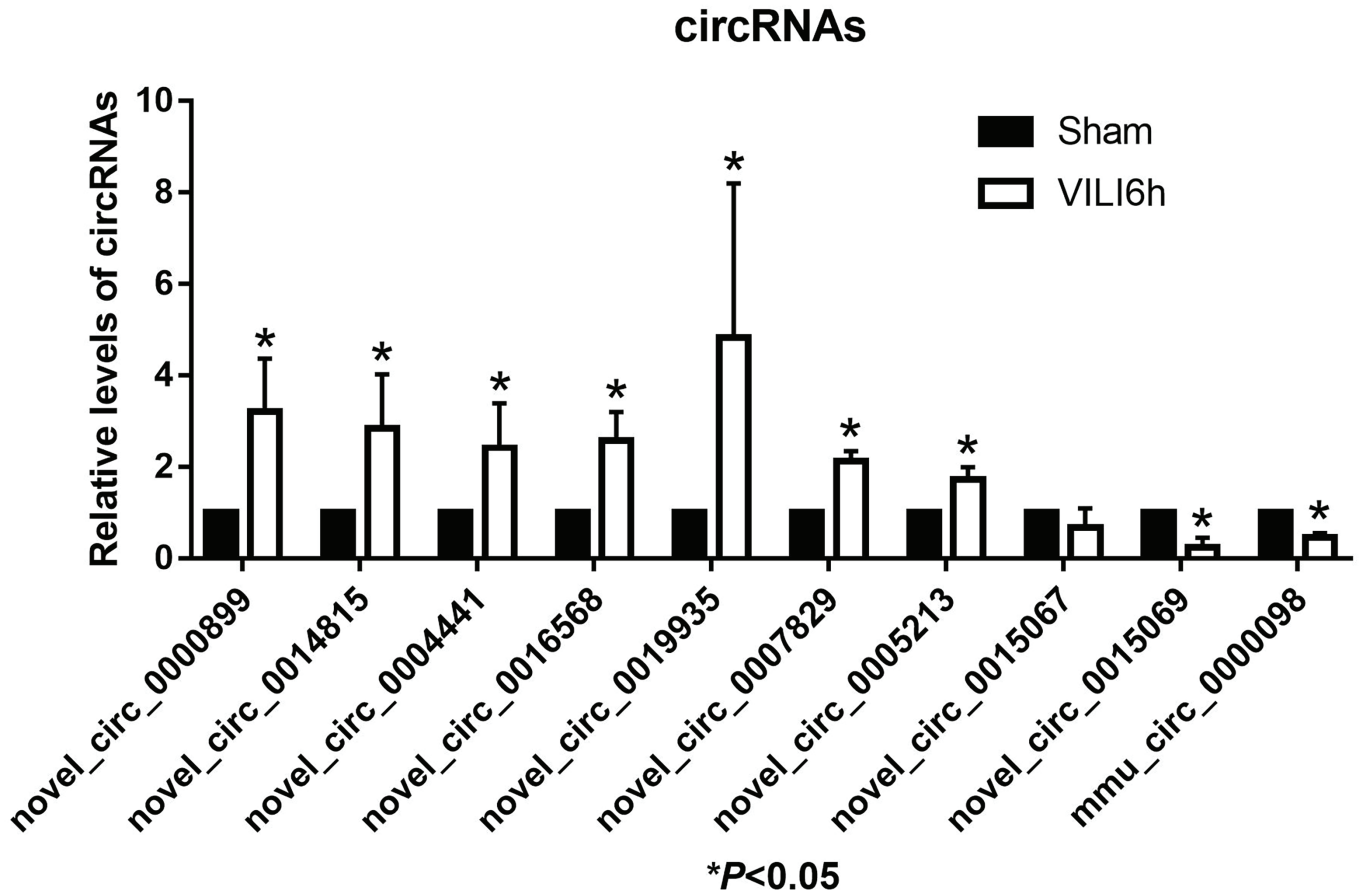
Validation of the top 10 differentially expressed circRNAs using qRT-PCR. Changes in differentially expressed circRNA were confirmed using qRT-PCR in the sham and VILI 6 h groups, *n* = 5. Bars represent means ± standard deviation. ^*^p < 0.05 VILI 6 h vs. sham.

The top 10 differentially expressed circRNAs, including seven upregulated circRNAs and three downregulated circRNAs, were further verified by qRT–PCR using mouse lung tissue from the VILI 6 h and sham groups. Compared to the sham group, the expression levels of novel_circ_0000899, novel_circ_0014815, novel_circ_0004441, novel_circ_0016568, novel_circ_0019935, novel_circ_0007829, and novel_circ_0005213 were upregulated in the VILI 6 h group (*p* < 0.05), and the expression levels of novel_circ_0015069 and mmu_circ_0000098 were downregulated (*p* < 0.05), which was basically consistent with the microarray results. However, there was no significant difference in the expression of novel_circ_0015067 between the two groups (*p* > 0.05). These results showed that most of the circRNAs identified by the microarray were reliable and worthy of further study (As shown in Figure 5).

### Identification of circRNA-Targeting miRNAs and Construction of circRNA-miRNA-mRNA Networks

**Figure 6:**
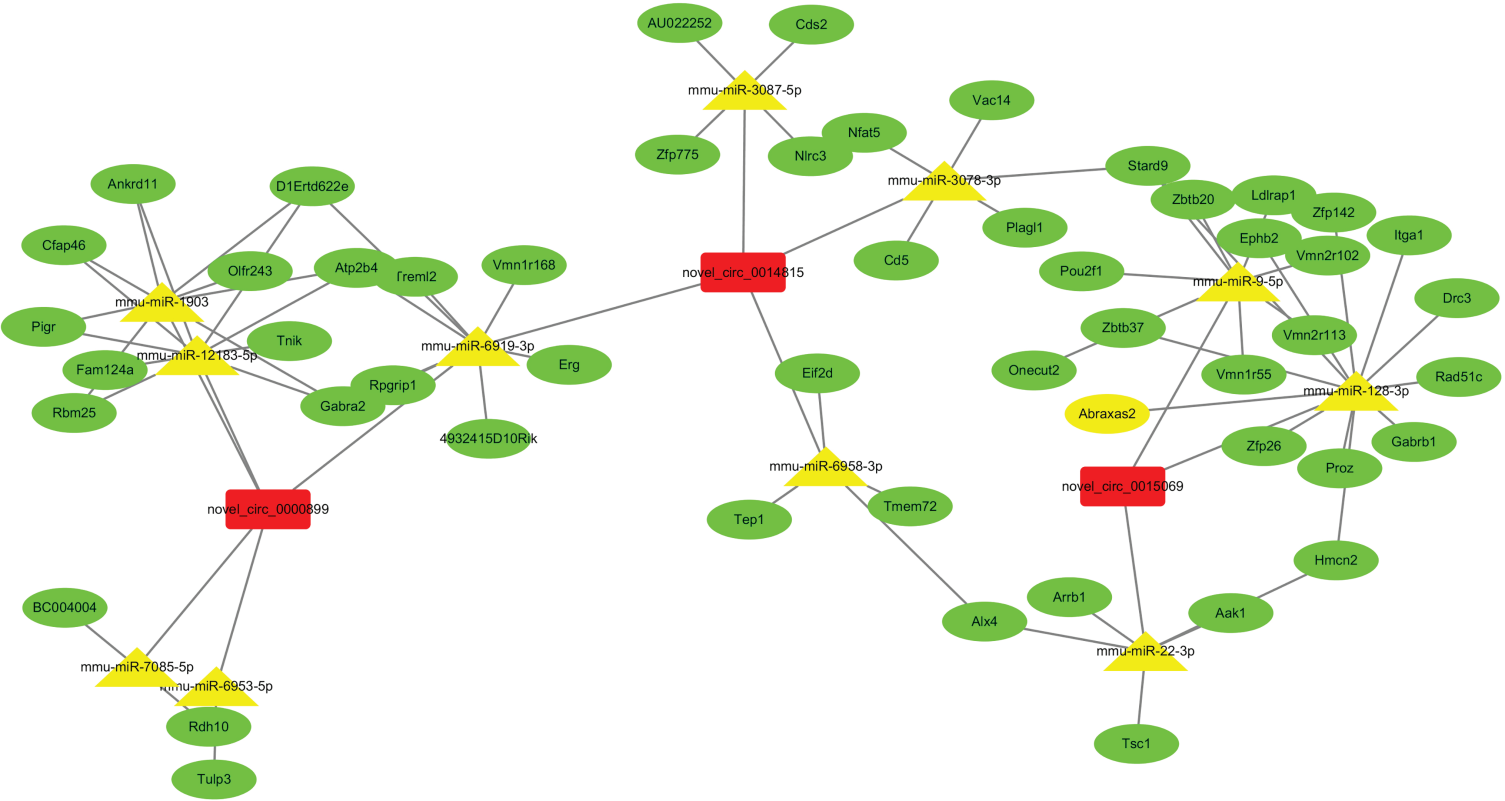
The network of ceRNA (circRNA-miRNA-mRNA). The two upregulated circRNAs and one downregulated circRNA were annotated in detail according to the circRNA-miRNA-mRNA interaction information from Cytoscape. Based on the miRNA and mRNA prediction, we showed that the top 5 miRNAs may be regulated by two upregulated circRNAs, one downregulated circRNA, and the top 30 target genes of each miRNA.

To better explore and predict the potential functions of differentially expressed circRNAs in the VILI 6 h model, we used miRanda to identify and verify the expression of novel_circ_0000899 and novel_circ_0014815, which exhibit the most significant upregulation, and novel_circ_0015069, which exhibits the most downregulation, to predict the target miRNAs and mRNAs. For each circRNA, we showed the top 5 miRNAs that may bind to it and the 30 target genes that were most likely to bind to each miRNA based on the target prediction score of the binding site according to the targeting relationship (As shown in Figure 6).

## Discussion

CircRNAs have a characteristic covalent closed loop structure and are conserved endogenous products with important functions. To date, however, there have been few reports on circRNA expression changes in VILI, and no circRNA regulatory network has been reported in VILI. In the present study, a total of 171 upregulated and 114 downregulated circRNAs were identified through microarray assays. Among them, the top 10 differentially expressed circRNAs were selected for validation using qRT-PCR. The verification results of nine circRNAs were consistent with the microarray results, and one circRNA was not significant. Our results demonstrated that changes in circRNA expression occurred after VILI in mice.

There are currently two main mechanisms to describe the onset and development of VILI. The first is that mechanical stretching directly damages the alveolar capillary basement membrane and extracellular matrix (ECM) and activates the related proteins and ion channels of the alveolar epithelium and vascular endothelial cell membrane mechanical sensitivity (Carrasco et al., 2015; Joshi and Morley, 2019). Subsequently, Toll-like receptors (TLRs) and the nuclear factor-κ B signaling pathway are triggered to induce inflammatory reactions (Carrasco et al., 2015; Joshi and Morley, 2019). The second mechanism is so-called “mechanical-biological conduction,” which converts mechanical stimulation into biological signal conduction when alveolar epithelium and vascular endothelial cells are mechanically stretched (Carrasco et al., 2015; Joshi and Morley, 2019). For example, high-tidal volume ventilation causes ECM remodeling, and mechanical conduction generates mechanical stress on the ECM, which causes lung strain and promotes the activation and release of matrix metalloproteinases, cytokines, and chemokines (Carrasco et al., 2015; Joshi and Morley, 2019). Briefly, long-term and excessive mechanical ventilation activates signal transduction, promotes reactive oxygen species production, releases inflamed mediators, and causes an inflammatory response, ultimately resulting in lung injury (Curley et al., 2016; Chen et al., 2018).

A large number of studies have confirmed that circRNAs mediate signal transduction pathways and regulate inflammation and oxidative stress, contributing to multiple diseases (Yang et al., 2017; Saaoud et al., 2021; Xue et al., 2021). Furthermore, circRNAs have been reported to play important roles in lung injuries induced by PM2.5 (Li et al., 2020), sepsis (Jiang et al., 2020; Zhou et al., 2021a), smoke (Zhou et al., 2021b), silica (Fang et al., 2018), or radiation (Li et al., 2021). In our study, we observed that there was differential expression of circRNAs in VILI. Therefore, we further predicted the possible mechanisms of circRNAs in VILI through bioinformatics analysis. GO analysis showed that circRNAs participated in a series of cellular processes, such as regulation of cellular macromolecule and protein metabolic processes, protein phosphorylation, chromatin modification, protein modification processes, chromatin organization, phosphorylation, and regulation of RNA metabolic processes. Previous studies have observed that circRNAs regulate oxidative stress and inflammation by affecting metabolism and protein phosphorylation (Chen et al., 2019; Saaoud et al., 2021). Therefore, we speculated that circRNAs may regulate oxidative stress and inflammation *via* metabolism and protein phosphorylation modification to lead to VILI. KEGG enrichment analysis showed that the Rap1 signaling pathway, B cell receiver signaling pathway, T-cell receiver signaling pathway, ECM receiver interaction, cGMP PKG signaling pathway, PI3K/Akt signaling pathway, and Ras signaling pathway were closely related to the differential expression of circRNAs in VILI mice. Among the identified pathways, activation of the Rap pathway and PI3K/Akt signaling pathway have been reported to have protective effects on VILI (Birukova et al., 2009; Li et al., 2013; Spassov et al., 2017; Ke et al., 2019). Therefore, we speculated that circRNAs may participate in the progression of VILI by regulating these signaling pathways. However, more in-depth research is needed to confirm our hypothesis in the future.

As a sponge to multiple miRNAs, circRNAs mediate target gene expression and contribute to cell functions *via* inhibition of miRNAs negatively regulating mRNA (Li et al., 2018; Kristensen et al., 2019; Chen, 2020). To further understand the possible mechanism, novel_circ_0000899, novel_circ_0014815, and novel_circ_0015069, which were the most significantly upregulated and downregulated circRNAs, were selected to predict the target miRNAs and mRNAs by miRanda. Our results showed that these three circRNAs had multiple miRNA binding sites. All of them regulated corresponding target genes *via* an endogenous competitive RNA mechanism. Unfortunately, these targeted prediction miRNAs have not been reported to play roles in VILI. Therefore, more research is needed to determine the relationship between circRNA-miRNA-mRNA networks and the development of VILI in the future.

## Conclusion

In summary, the present study identified the circRNA expression profile of lung tissue from mice with VILI. The results provide new ideas for the identification of new mechanisms of VILI and lay a foundation for the screening and prevention of targets of circRNAs as new markers of VILI.

## Data Availability Statement

The datasets presented in this study can be found in online repositories. The names of the repository/repositories and accession number(s) can be found below: National Center for Biotechnology Information (NCBI) BioProject database under accession number PRJNA798173.

## Ethics Statement

The studies involving mice were approved by the Research Ethics Committee of China-Japan Friendship Hospital.

## Author Contributions

SC, JX, and YZ participated and conceived the study design. SC and JX collected the data. SC performed the experiments, analyzed the data, and wrote the manuscript. SC, QZ, and YZ interpreted and discussed the data. QZ and YZ refined the final draft and revised the manuscript. All the authors reviewed the final version of the manuscript.

## Funding

This study was supported by grants from the National Natural Science Foundation of China (no: 81870072) to QZ.

## Conflict of Interest

The authors declare that the research was conducted in the absence of any commercial or financial relationships that could be construed as a potential conflict of interest.

## Publisher’s Note

All claims expressed in this article are solely those of the authors and do not necessarily represent those of their affiliated organizations, or those of the publisher, the editors and the reviewers. Any product that may be evaluated in this article, or claim that may be made by its manufacturer, is not guaranteed or endorsed by the publisher.

## Footnotes

^1^ http://www.genome.jp/kegg/
