# Supplementary material for: Microarray Analysis Reveals the Changes in Circular RNA Expression and Molecular Mechanisms in Mice With Ventilator-Induced Lung Injury
Source: Front Physiol. 2022 Mar 10;13:838196. doi: 10.3389/fphys.2022.838196 (PMC8960733; doi:10.3389/fphys.2022.838196)
Supplement: Supplementary file 1 [file Presentation_1.pdf]

Supplementary material

SUPPLY FIGURE 1 Classification of Raw Reads

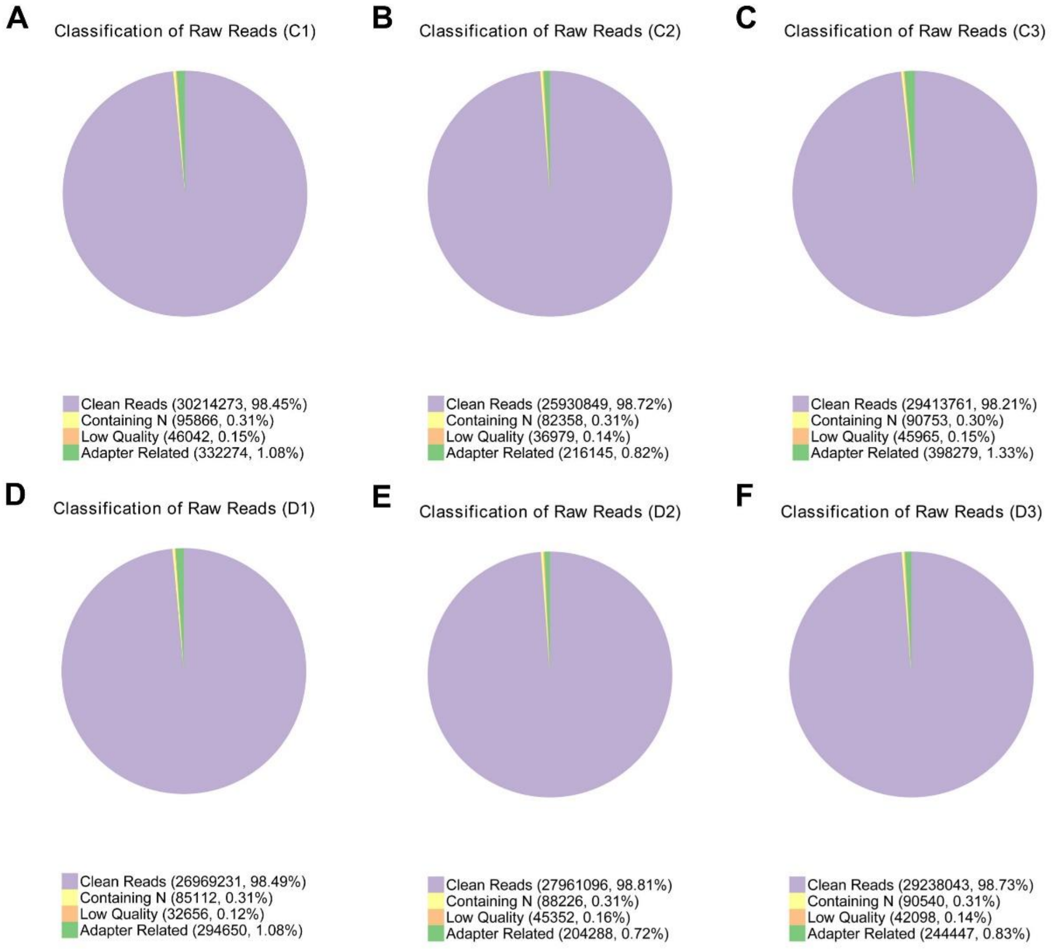

SUPPLY FIGURE 2 Distribution of sequencing error rate

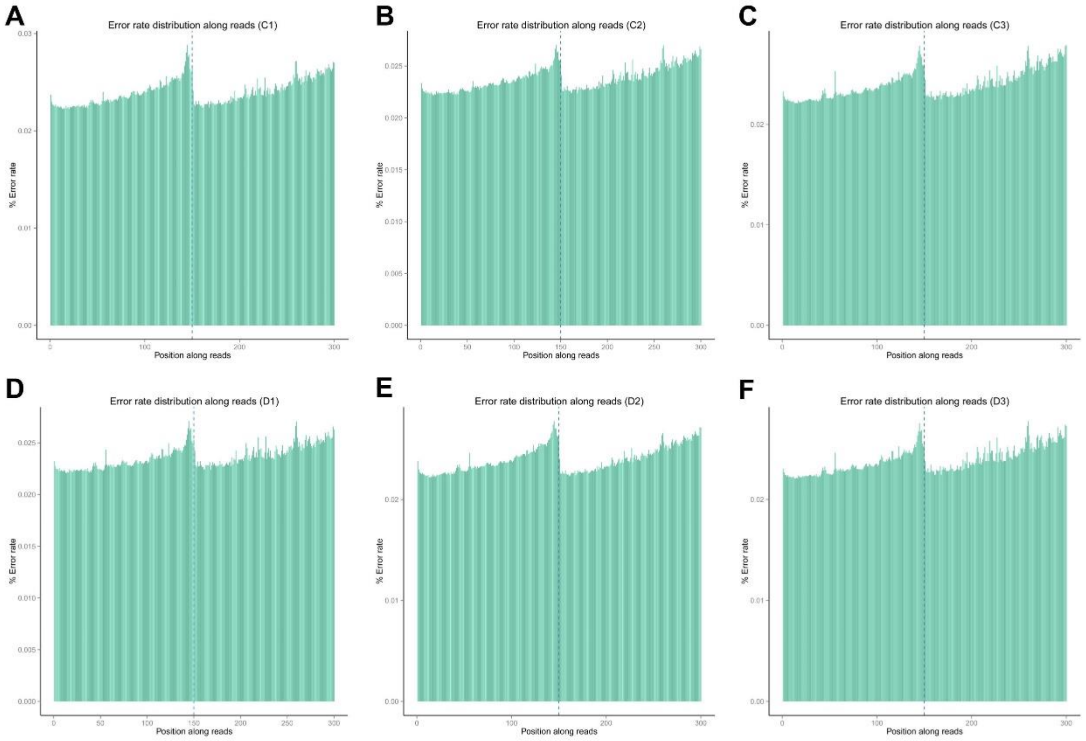

SUPPLY FIGURE 3 GC content distribution

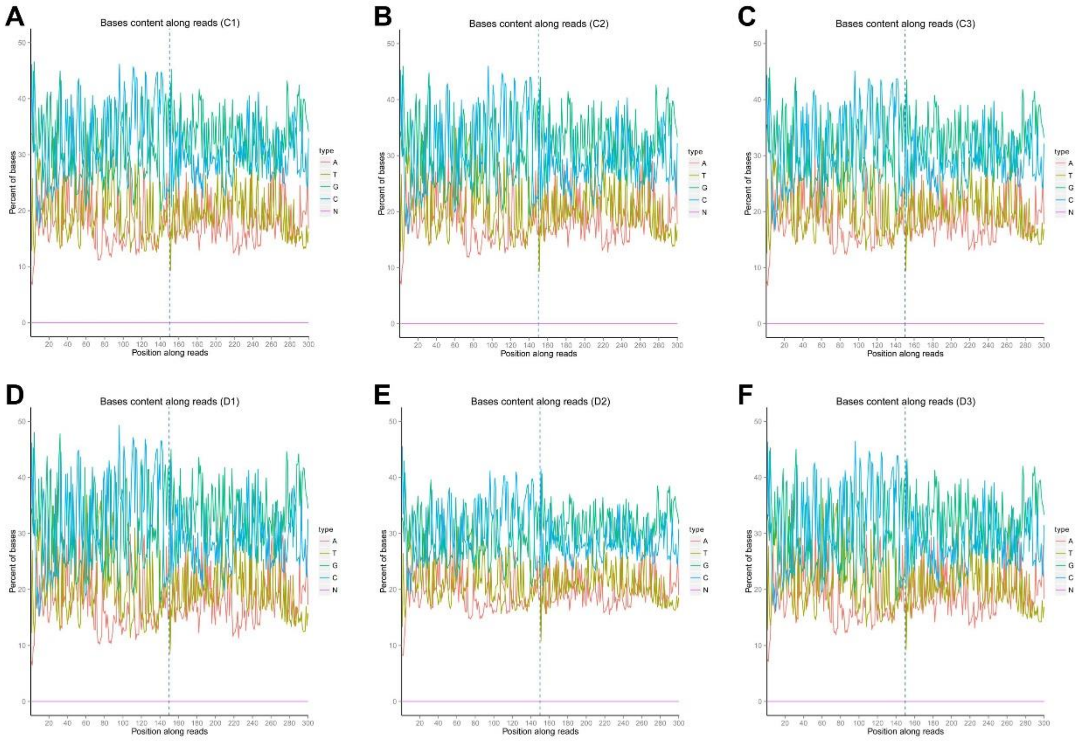

SUPPLY FIGURE 4 Mapping Region of Clean Reads

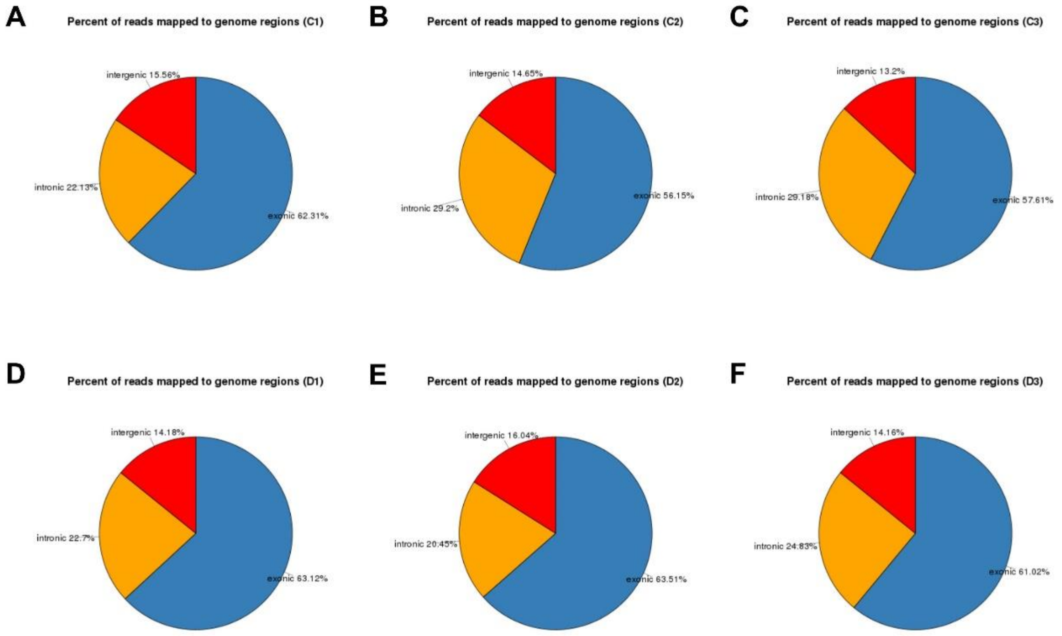

SUPPLY FIGURE 5 CircRNAs chromosome distribution

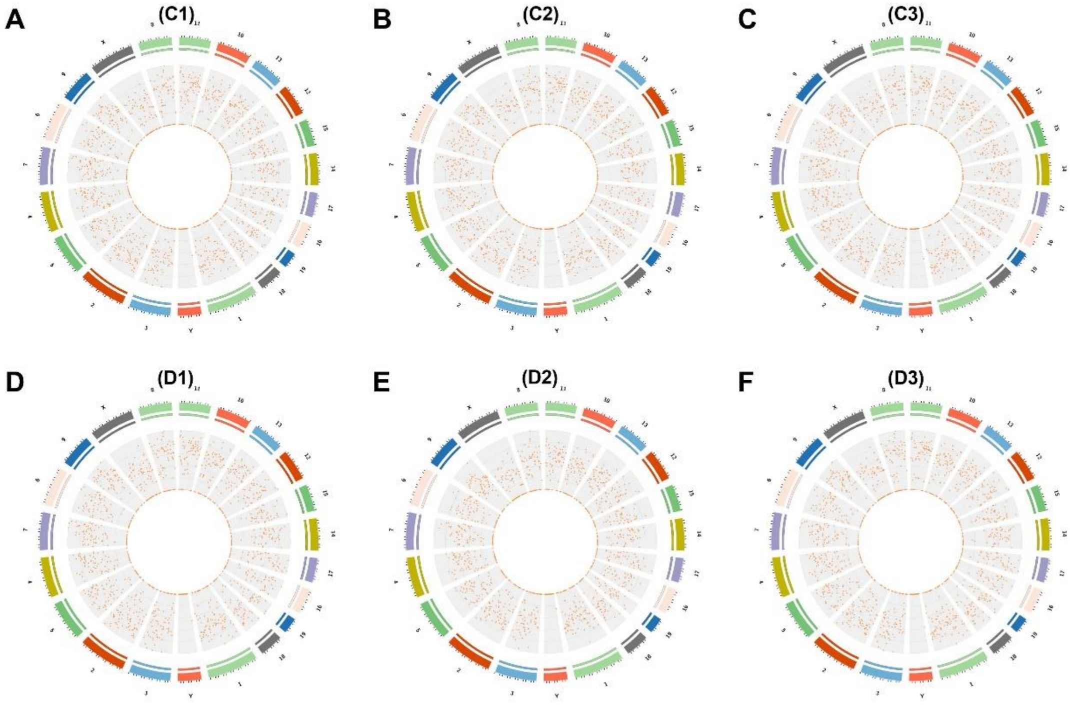

SUPPLY FIGURE 6 CircRNAs source statistics

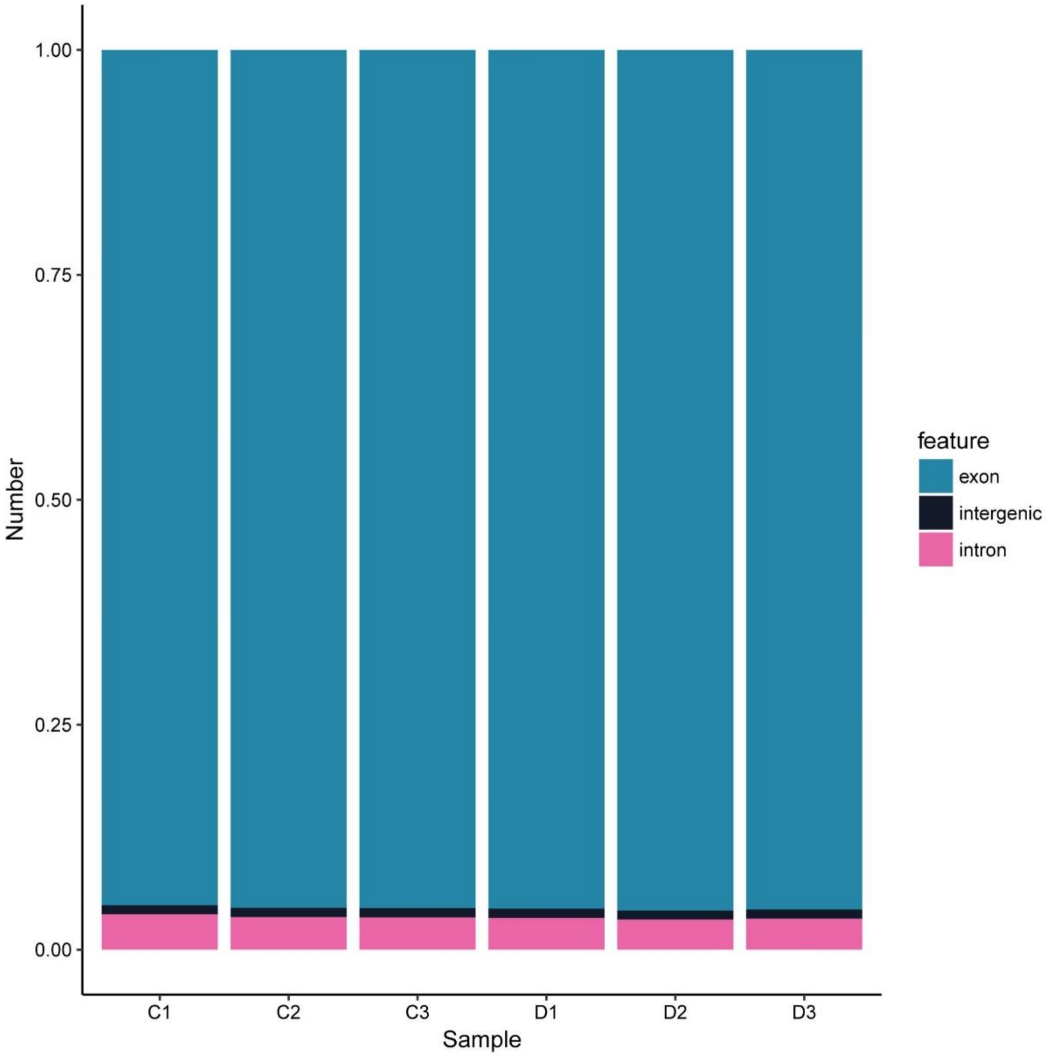

SUPPLY FIGURE 7 CircRNAs length distribution

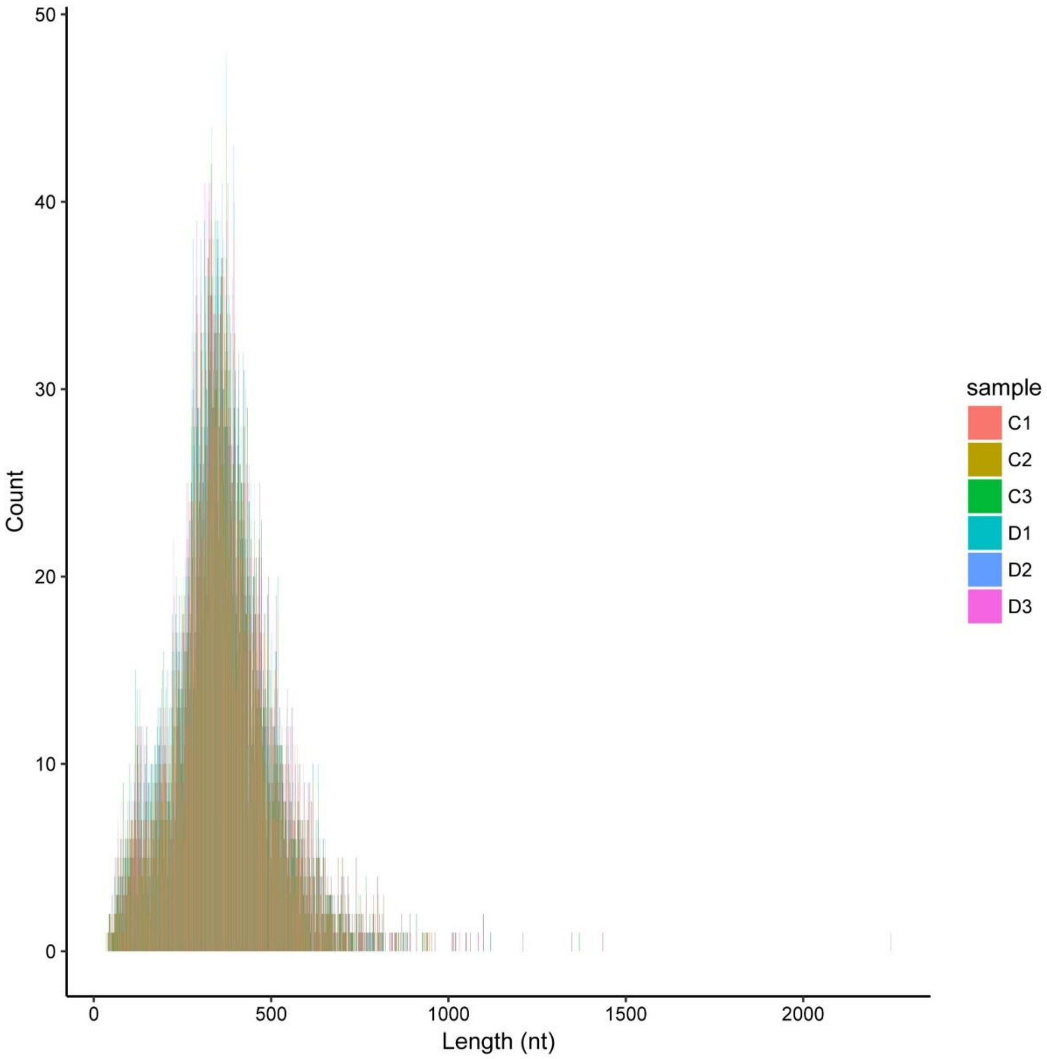

SUPPLY TABLE 1 Clean reads

Sample_name	Raw_reads	Clean_reads	Raw_bases(G)	Clean_bases(G)	Error rate(%)	Q20(%)	Q30(%)	GC_content(%)
C1	61376910	60428546	9.21	9.06	0.02	98.34	95.28	62.54
C2	52532662	51861698	7.88	7.78	0.02	98.45	95.48	60.86
C3	59897516	58827522	8.98	8.82	0.02	98.36	95.28	61.4
D1	54763298	53938462	8.21	8.09	0.02	98.53	95.62	61.77
D2	56597924	55922192	8.49	8.39	0.02	98.37	95.29	59.37
D3	59230256	58476086	8.88	8.77	0.02	98.43	95.35	59.94

(1) Sample name.

(2) Raw reads, the number of reads in the raw data.

(3) Clean reads, the number of reads after filtering the original data.

(4) Raw bases, the number of bases in the raw data.

(5) Clean bases, the number of bases after filtering the original data.

(6) Error rate, overall data sequencing error rate.

(7) Q20, The percentage of bases with a Phred value greater than 20 to the total bases.

(8) Q30, The percentage of bases with a Phred value greater than 30 to the total bases.

(9) GC content, the percentage of G and C in the four bases in clean reads.

SUPPLY TABLE 2 Sample comparison

Sample name	C1	C2	C3	D1	D2	D3
Total reads	60428546	51861698	58827522	53938462	55922192	58476086
Total mapped	50027732 (82.79%)	44815944 (86.41%)	50176215 (85.29%)	48334971 (89.61%)	47568706 (85.06%)	53077175 (90.77%)
Multiple mapped	21051307 (34.84%)	18674199 (36.01%)	19866597 (33.77%)	22604494 (41.91%)	14609967 (26.13%)	22537712 (38.54%)
Uniquely mapped	28976425 (47.95%)	26141745 (50.41%)	30309618 (51.52%)	25730477 (47.7%)	32958739 (58.94%)	30539463 (52.23%)
Read-1	14520777 (24.03%)	13101191 (25.26%)	15194174 (25.83%)	12896643 (23.91%)	16527340 (29.55%)	15313454 (26.19%)
Read-2	14455648 (23.92%)	13040554 (25.14%)	15115444 (25.69%)	12833834 (23.79%)	16431399 (29.38%)	15226009 (26.04%)
Reads map to '+'	14488082 (23.98%)	13071598 (25.2%)	15155111 (25.76%)	12862630 (23.85%)	16469946 (29.45%)	15263851 (26.1%)
Reads map to '-'	14488343 (23.98%)	13070147 (25.2%)	15154507 (25.76%)	12867847 (23.86%)	16488793 (29.49%)	15275612 (26.12%)
Non-splice reads	22658591 (37.5%)	20818023 (40.14%)	24168947 (41.08%)	20381051 (37.79%)	23650243 (42.29%)	23645045 (40.44%)
Splice reads	6317834 (10.46%)	5323722 (10.27%)	6140671 (10.44%)	5349426 (9.92%)	9308496 (16.65%)	6894418 (11.79%)
Reads mapped in p	28073954 (46.46%)	25307242 (48.8%)	29347548 (49.89%)	24957794 (46.27%)	32063006 (57.34%)	29653366 (50.71%)
Proper-paired read	0 (0%)	0 (0%)	0 (0%)	0 (0%)	0 (0%)	0 (0%)

(1) Sample name.

(2) Total reads, the number of clean reads used for comparison analysis.

(3) Total mapped reads, The number of reads aligned to the genome.

(4) Multiple mapped reads, The number of reads aligned to multiple positions in the genome.

(5) Uniquely mapped reads, The number of reads aligned to a single location in the genome.

(6) Read-1, The number of reads aligned to the genome in read1.

(7) Read-2, The number of reads aligned to the genome in read2.

(8) Reads map to'+', The number of reads aligned to the positive strand of the genome.

(9) Reads map to'-', The number of reads aligned to the negative strand on the genome.

(10) Non-splice reads: The number of reads compared to exons in the whole segment.

(11) Splice reads, The number of reads (also called Junction reads) that were aligned to two exons. The percentage of Splice reads depended on the length of the sequenced fragment.

(12) Reads mapped in proper pairs, The percentage of reads mapped in the reference genome to the total number of clean reads.

(13) Proper-paired reads map to different chrom, The percentage of reads that were aligned to different chromosomes on the genome to the total number of clean reads.

C and D represent sham and VILI6h groups, respectively
